# Happiness in marginalized populations: a community–based study in South Central Iran

**DOI:** 10.1186/s40359-021-00545-2

**Published:** 2021-04-23

**Authors:** Maryam Kazemi, Behnam Honarvar, S. Taghi Heydari, Hassan Joulaei, Mohammad Reza Rahmanian Haghighi, Kamran Bagheri Lankarani

**Affiliations:** Health Policy Research Center, Institute of Health, Shiraz University of Medical Sciences, Shiraz, 7134845794 Islamic Republic of Iran

**Keywords:** Happiness, Well–being, Slum area, Marginalized populations, Iran

## Abstract

**Background:**

Happiness has multiple levels and determinants in different communities, cultures, and social groups. The current study aimed to investigate happiness and its main determinants in slums in south central Iran.

**Methods:**

This community-based, cross-sectional study was conducted with the participation of adults at least 18 years of age living in the biggest slum area in Shiraz, south central Iran. To determine levels of happiness, participants were asked to complete the Persian version of the GHQ28 questionnaire and a checklist based on the 2017 World Happiness Report. Data was analyzed using SPSS software version 19. A *p*-value less than 0.05 was considered significant.

**Results:**

The mean age of the participants was 42.06 ± 16.34 years. Overall, 542 participants (45 %) were females, 257 (21.3 %) were illiterate, 678 (56.3 %) were married, and 495 (41.1 %) were unemployed and lived with their household. The happiness score, according to the Cantril ladder score, was 6.41 ± 2 (out of a total score of 10). Happiness was not correlated with gender (*p* = 0.37) or immigration (*p* = 0.06). Lower levels of happiness were seen in older adults (r=− 0.12, *p* < 0.001), illiterates (*p* = 0.03), the unemployed (*p* < 0.001), and people separated from their spouses (*p* < 0.001). Job satisfaction (*p* < 0.001, r = 0.47), total general health (*p* < 0.001, r=-0.36) and hope (*p* < 0.001, r = 0.41) were significantly correlated with happiness. Social support (< 0.001) and sufficient income and satisfaction (*p* < 0.001) were related with a higher score of happiness.

**Conclusions:**

Marital status, smoking, employment and job satisfaction, social support and trust, feelings of insecurity in the neighborhood, hope for the future, facing violence, and income satisfaction were the main determinants of happiness in the Sang Siah slum area.

**Supplementary Information:**

The online version contains supplementary material available at 10.1186/s40359-021-00545-2.

## Background

Happiness, influenced by socioeconomic status, physical and mental health, social status, and social interrelationships, has become an interesting topic for policy making in recent years [1]. Veenhoven defined happiness as the degree to which an individual judges favorably the overall quality of his/her own life as a whole [2].

Happiness is known as a measure of social progression and is used as a goal for policy making in different countries. In 2011, the Organization for Economic Co-operation and Development (OECD) put citizens’ happiness in the core of governments’ focus [2]. Happiness is an important factor for the development of a country due to its correlation with employment, job security, and income [3], and it increases with economic growth in nations [4].

Average happiness varies in different countries and communities, possibly because of differences in social characteristics [5] and good living standards [6]. Differences in culture are important factors in determining the differences in levels of happiness [7]. The highest and lowest scores for happiness can be seen in northern and central Europe and Asian countries with transitional economies, respectively [8].

OECD identified 11 topics, i.e. housing, income, job, community, education, environment, civic engagement, health, life satisfaction, safety, and life-work balance, as Better Life Indices [2]. Moreover, six important factors have been mentioned in happiness reports as the main reasons for different levels of happiness in different regions and countries, comprising GDP per capita as all the values of services and goods produced by a country over the time divided by the population number, healthy years of life expectancy, social support as measured by having someone to count on in times of trouble, trust as measured by a perceived absence of corruption in government and business, perceived freedom to make life decisions, and generosity as measured by recent donations [9].

With the fast growth of urbanization seen today, populations in slums are growing in developing countries like Iran [10]. According to the United Nations definition of slums based on Sustainable Development Goals (SDG), “A slum household is defined as a group of individuals living under the same roof lacking one or more of the following conditions: access to improved water, access to improved sanitation, sufficient living area, and durability of housing” [11].

Poverty in slums causes many social problems, including crime and addiction [12]. People living in slums have inappropriate sanitation and healthcare access that affect their lives, health [13, 14], and life satisfaction [15]. Basic needs assessments of such communities should be performed to plan and employ interventions to improve life standards and happiness that will reflect on the health of these communities. To the best of our knowledge, no study has investigated happiness and its determinant factors in slum areas in Iran. The results of this study will provide policy makers with interventions that could be designed to improve the health and happiness of people living in the slums of Iran.

## Methods and materials

### Study design

This cross-sectional study was conducted in 2017–2018 on adults living in the Sang Siah neighborhood of Shiraz, located in south-central Iran. Known as the main slum neighborhood of Shiraz, Sang Siah is located in the old town texture of Shiraz and has a population of approximately two million.

### Sampling

The postal codes of the Sang Siah neighborhood were taken from the Central Post Office. All adults 18 years of age or higher and living in a house were registered and asked to answer some questions. Personal interviews with each participant were conducted based on the municipal map and postal codes of the neighborhood. Trained interviewers visited households in front of their house (respecting the occupants’ privacy) and asked them one by one to answer questions without the presence of another family member. The surveys were completed during a period of several months using tablet devices. Participants first received an explanation of the aims of the study and assured of the confidentiality of the information disclosed by them. Each participant then signed a consent form. If a resident was not at home, the interviewer referred again; if that resident did not show up for the interview after two more visits, s/he was excluded from the study.

### Study instruments and variables

One checklist and a questionnaire were used to collect data. The checklist included 27 questions. In the first part, demographic characteristics of participants such as age, gender, marital status, job, education level, and income (whether they had enough income for their living costs) were asked. Questions about immigration, current hookah and/or cigarette smoking, alcohol consumption, frequency of opium usage during the preceding year, number of children, insurance status, and any affliction with a chronic disease were also asked.

In the second part of checklist, participants were asked about their life satisfaction (Satisfaction with Life scale with five ways) [16], job satisfaction, and neighborhood safety (crimes in the neighborhood). A visual ladder scale from 0 to 10 was used to report their answers. Hope for the future was scored from − 5 to + 5 and measured with a visual ladder scale.

To evaluate happiness as a dependent variable in this study, the Cantril Ladder score (0 to 10) was used, which is a standard method [17]. People were asked to imagine a ladder with steps numbered from 0 at the bottom to 10 at the top. The top of the ladder represents the best possible life, and the bottom represents the worst possible life. Then they were asked on which step of the ladder they felt they stood at this moment [9]. To evaluate social support, participants were asked if they were in trouble and if they had relatives or friends they could count on for help whenever needed [9].

Participants were asked about their religious beliefs and performance of religious duties (praying and fasting for Muslims) using a 5-point Likert scale (from completely to not at all). They were asked if they had been violent or exposed to at least one type of physical, sexual, or emotional violence at home or outside the home with four questions. Participants were also asked if they or their relatives and/or friends had noticed bribery by government officers in the past [9]. Social support was measured by asking if they had someone to count on in times of trouble. The checklist of questions and response options of the variables are presented in Additional file 1: Appendix 1.

The general health of participants was evaluated with the validated Persian translation of the General Health Questionnaire (GHQ) comprised of 28 questions, including 7 each about depression, anxiety and insomnia, social dysfunction, and somatic symptoms. A 4-point Likert scale (from 0 to 3) was used [17].

### Statistical analysis

Data was analyzed using IBM SPSS statistics software version 19. Descriptive analysis (including mean, standard deviation, median and frequency), independent t-test (for two continuous variables), chi-square test (for categorized variables), one-way ANOVA (for three or more continuous variables), Pearson’s correlation (for the correlation of two continuous variables), and linear regressions model - backward method were also used to explain the factors that affect the average of happiness. A *p*-value of < 0.05 was considered significant.

## Results

From 1475 people at least 18 years of age and living in Sang Siah, 1204 people participated in this study, a response rate of 81.6 %.

The mean age of the participants was 42.06 ± 16.34 years. In this neighborhood, 662 participants (55 % of the sample) were males. The level of education in 531 participants (44.1 % of the sample) was primary school, and 678 participants (56.3 % of the sample) were married. The Median number of children in families was 3 (mean ± SD; 2.95 ± 1.98), and 279 participants (23.2 % of the sample) had immigrated from other cities, villages, or countries. Of the study participants, 495 (41.1 % of the sample) were unemployed housewives, and 946 (78.6 %) reported that their living expenses were higher than their income (Table 1).

**Table 1:** Socio-demographic characteristics of participants

Variable	Amount	Variable	Amount
Age (year)		Job *n* (%)	
Mean ± SD	42.06 ± 16.34	Unemployed	124 (10.3 %)
Min–Max	18–99	Housewife	371 (30.8 %)
Gender n(%)		Student	52 (4.3 %)
Male	662( 55 %)	Daily worker	319 (26.5 %)
Female	542 (45 %)	Manual worker	32 (2.7 %)
Level of education n (%)		Employee	25 (2.1 %)
Illiterate	257 (21.3 %)	Manager	94 (7.8 %)
Primary school	531 (44.1 %)	Retired	97 (8.1 %)
Diploma	308 (25.6 %)	Disable	25 (2.1 %)
Associate degree and bachelor	102 (8.5 %)	Income/ cost n (%)	
Master and PHD	6 (0.5 %)	> 1	0 (%)
Marital status n (%)		< 1	946 (78.6 %)
Single	361 (30 %)	= 1	258 (21.4 %)
Married	678 (56.3 %)	Immigration n (%)	
Divorced	47 (3.9 %)	Yes	279 (23.2 %)
Separated	21 (1.7 %)	No	925 (76.8 %)
Widowed	97 (8.1 %)		

The results showed that 488 participants (40.5 % of total sample) had no insurance, 401 (33.3 % of sample) had at least one chronic disease for which they took medication or were referred to physicians.

The general health score was categorized as: 0 to 22 (no dysfunction), 23 to 40 (mild dysfunction), 41 to 60 (moderate dysfunction), and 61 to 84 (severe dysfunction) [18]. For each subgroup of general health, a score of 0 to 6 is representative of no dysfunction. A score of 7 to 11, 12 to 16, or 17 to 21 shows mild, moderate, or severe dysfunction, respectively.

The total score of general health of people, according to the GHQ, was 18.5 ± 8.59, which implies that the participants’ general health was within the normal range. The mean score of social dysfunction was 7.54 ± 3.04, indicating mild social dysfunction. The mean scores of somatic symptoms, anxiety and insomnia, and depression were 4.74 ± 2.88, 4.32 ± 3.55, and 1.89 ± 3.04, respectively, which showed no dysfunction.

As self-reported by the participants, 33 (2.7 %) were alcohol users, 53 (4.4 %) used at least one kind of opium, 113 (9.4 %) were hookah smokers, and 214 (17.8 %) were cigarette smokers. As religious beliefs and religious duties were concerned, 544 of the participants (45.2 %) said they were strictly religious, and 395 of them (32.8 %) observed all their religious duties.

 Moreover, 556 of the study participants (46.2 %) reported that they have someone (friend, relative, or neighbor) to help them in emergency situations, and 729 of the participants (60.5 %) said they have someone upon whom they could rely and with whom they could consult. After explaining all types of violence (physical, emotional, and sexual), the participants were asked about their experience with it. As a result, 143 participants (11.9 %) had been subjected to violence, and 71 (5.9 %) had exhibited violent behavior toward someone else. Of all the participants, 169 people (14 %) reported having witnessed bribery by officials during their visits to government offices in the preceding year.

The mean scores for happiness, life satisfaction, income, and job satisfaction were 6.41 ± 2, 6.37 ± 1.8, 4.63 ± 2.12, and 5.73 ± 2.12 out of 10, respectively. The average score for hope was 2.91 ± 1.97.

The correlations between happiness and socio-demographic characteristics are shown in Table 2. As can be seen, happiness was not significantly different between males or females (*p* = 0.37) or in immigrants and other groups of people (*p* = 0.06). Higher scores of happiness were seen in higher educated people (*p* = 0.03), singles (*p* < 0.001), and people with enough income to meet their living costs (*p* < 0.001). Participants who were disabled had the lowest score of happiness (*p* < 0.001). The results showed that age had a negative and significant correlation with happiness (r=-0.12, *p* < 0.001).

**Table 2:** Relationship between happiness and socio-demographic characteristics of participants

Variable	Happiness Mean±SD	*p* Value	Variable	Happiness Mean±SD	*p* Value
Gender			Job		
Male	6.37±1.92	o.37	Unemployed	5.91 ± 1.93	< 0.001
Female	6.47±2.09		Housewife	6.51 ± 2.12	
Level of education			Student	7.17 ± 1.13	
Illiterate	6.24±1.88	0.03	Daily worker	6.54 ± 1.66	
Primary school	6.3±2.12		Manual worker	6.47 ± 2.63	
Diploma	6.62±1.81		Employee	6.28 ± 2.5	
Associate degree and bachelor’s degree	6.79±1.93		Manager	6.52 ± 1.9	
Master ‘s degree and above	6.5±3.93		Retired	6.63 ± 1.99	
			Disable	4.36 ± 2.49	
Marital status			Income/cost		
Single	6.72±1.7	<0.001	>1	–	< 0.001
Married	6.45±2.02		<1	6.23 ± 2.04	
Divorced	5.26±2.48		=1	7.07 ± 1.66	
Separated	4.81±2.76		Immigration		
Widowed	5.86±1.99		Yes	6.2 ± 2.19	0.06

Happiness had a negative and significant correlation with total general health and all aspects of the general health of the people, including somatic symptoms, anxiety and insomnia, social dysfunction, and depression (*p* < 0.001).

Satisfaction with life, hope for the future, job satisfaction, and safety in the neighborhood had significant positive relationships with happiness (*p* < 0.001). Mean happiness in people who have a friend or family member to talk to was 6.68 ± 1.96, which was significantly higher than in those without friends or family (6 ± 1.98 (*p* < 0.001).

People who have someone to help them in emergency situations were happier than others (6.95 ± 1.73 compared with 5.94 ± 2.09, respectively). Happiness was significantly lower in people who had been subjected to violence (4.99 ± 2.65) compared with others (6.6 ± 1.81). Those participants who had observed bribery among government officials during the previous year had a significantly lower mean score for happiness (5.91 ± 2.5) than others (6.49 ± 1.89). Cigarette and hookah smokers were less happy than non-smokers (*P* < 0.001), but there was no difference between the happiness levels of alcohol consumers and non-consumers (*p* = 0.05). Opium users were significantly less happy than non-users (*p* < 0.001). Participants with religious beliefs were happier than others (*p* = 0.001).

All variables which were correlated with happiness in univariate analysis at a significant level of < 0.2 were added to a linear regression model (Table 3). The main determinants of happiness in Sang Siah were those with a *p-*value < 0.05 in the model.

The linear regression model can predict happiness correctly at 54 % (adjusted R^2^ = 0.54).

**Table 3:** Linear regression model for predicting happiness in slums of Shiraz, Iran

*Variable	β	SE	t	95 % CI	*p* Value
Constant	1.55	0.95	1.62	− 0.32-3.43	0.1
Marital status	− 0.3	0.07	− 3.94	− 0.45- -0.15	< 0.001
Job satisfaction	0.15	0.03	4.57	0.08–0.22	< 0.001
Having job	− 0.12	0.04	− 2.96	− 0.21- -0.04	0.003
Social support	− 0.34	0.12	− 2.64	− 0.59- -0.08	0.008
Hope to the future	0.1	0.03	3.14	0.04–0.17	0.002
Life satisfaction	0.18	0.04	4.4	0.1–0.26	< 0.001
Cigarette smoking	0.22	0.13	1.65	− 0.04- -0.49	0.09
Opium usage	− 0.69	0.29	− 2.33	− 1.27- -0.1	0.02
Violence in neighborhood	0.45	0.12	3.66	0.21–0.69	< 0.001
Home satisfaction	0.08	0.02	2.83	0.02–0.13	0.005
Income satisfaction	0.21	0.03	5.53	0.13–0.28	< 0.001
Migration	0.29	0.12	2.37	0.05–0.54	0.01
Religious believes	0.13	0.05	2.36	0.05–0.19	0.001
Mental disease	0.12	0.03	3.45	0.05–0.19	0.001
Chronic disease	0.09	0.04	2.17	0.009–0.17	0.03

*Marital status: 1-Single, 2- Married 3- Divorced,4-Separated,5-Widowed, Job satisfaction: 0 to 10, Having job: 1-Yes, 2- No, Social support: 1-Yes, 2-No, Hope to the future:0 to 10, Life satisfaction:0 to 10, Cigarette smoking:1- Yes,2- No, Opium consumption: 1-Yes, 2- No, Violence in neighborhood:0 to 10, Home satisfaction:0 to 10, Income satisfaction:0 to 10, Migration: 1-Yes,2- No, Religious believes:1- not at all, 2-sometimes,3- Often, 4- Always, 5- completely, Mental disease: 1-Yes, 2-No, Chronic disease: 1-Yes, 2-No

## Discussion

The current findings showed that the level of happiness was not lower in the slums than among all Iranians [19]. Happiness was not different in men compared to women. Lower levels of happiness were observed in elderly, illiterate, unemployed, and separated people. Job satisfaction, life satisfaction, hope, social support, and sufficient income to cover living expenses were positively correlated with the happiness score.

In this study, the mean score of happiness in a slum area of Shiraz (Sang Siah) was 6.41. The mean score of Iranian happiness was reported to be 5.08 in 1997 [19]. In the 2017 World Happiness Report, Iran was ranked 108th with a score of 4.69 [9]. The World Happiness Score may not accurately reflect the population’s social well-being and happiness. Nationwide or citywide studies are needed to better understand the city or nation’s well-being. According to the theory of adaptation, having recently repeated negative stimuli, such as the effects of the economic sanctions on Iran, plays an important role in understanding happiness and social well-being and can result in different perceptions of good living standards by different people [20].

In this study, happiness was higher in single and married participants than in separated, divorced, or widowed ones. However, in a cross-national study on 39,082 participants from 29 Asian countries, it was determined that married participants were happier than single ones [21]. A longitudinal study by Stutzer reported that happier single people get married sooner than other groups and that married people are not happier than single ones [22]. Divorce or death of a spouse is correlated with psychological distress [23] and hopelessness [24]. In Iranian culture, divorce is unpopular and makes one vulnerable [25], and it could be the cause of unhappiness.

It was also determined that with an increase in job satisfaction, happiness scores increased, and unemployed participants had the lowest level of happiness. Some studies have shown that job satisfaction is a more important factor for happiness than having a job [26, 27]. Although Clark mentioned that unemployment had a greater effect on one’s well-being than financial satisfaction with one’s job [28], the current study shows that both employment and job satisfaction are among the important factors for well-being. Clark argues that in a region with a high rate of unemployment, people notice many other jobless people and therefore, they do not blame themselves for being unemployed, and having no job has a lesser effect on their wellbeing [28].

The results showed that people with no social support had lower scores of happiness. This is in line with the results of Hart et al. on 6037 participants in a SPOTLIGHT survey with a large social network in which trusted neighbors were correlated with higher levels of happiness [29]. In another study on South Korean university students, happiness was correlated with their perception of social support [30].

In the slums of Calcutta, social well-being was lower than the other groups compared, but it was higher than expected due to strong social relationships [15]. Another study in Nicaragua showed negative subjective well-being in people living in marginalized urban areas, and the most predictive factors affecting subjective well-being were objective income and social support [31].

The current results showed that life satisfaction is significantly correlated with happiness. Other studies have shown that these two variables are different in concept. Happiness depends on country characteristics and people living in a stable relationship, but life satisfaction is related to country of living and feelings of control [32].

Hope for the future had a positive correlation with happiness and could be predicted in this study. Other investigators have shown that happiness and hope are correlated with each other, but this correlation is not positive in all studies; some investigators have demonstrated a negative association between happiness and hope [33]. In a study on university students, however, a positive correlation between hope and subjective well-being was demonstrated [34].

Cigarette smokers were less happy than non-smokers in Sang Siah neighborhood, but in the presence of other factors, cigarette smoking was not correlated with happiness. This means that confounders such as socioeconomic factors affect this relation. A study on smoking status and happiness in nine countries of the former Soviet Union (fSU) showed that ex-smokers were happier than smokers [35].

According to the current results, opium-addicted participants were happier than others, probably due to mood changes in opium users [36]. However, this could be related to the dose and duration of consumption, which was not evaluated in this study. Happiness did not differ between participants consuming and those not consuming alcohol. It seems that the relationship between happiness and alcohol consumption is related to the type of alcohol and the culture of the people [37].

The current results indicated that 11.9 % of the participants had been subjected to violence. In a systematic review in Iran, the prevalence of domestic violence was reported to fluctuate between 5.4  and 94.7 % [38]. Participants who faced violence at home or in the neighborhood were less happy than others. The current results agree with the findings of the study which evaluated contextual correlates of happiness in European adults that feeling safety in a neighborhood was associated with higher levels of happiness [29]. A review of domestic violence in Iran showed that violence causes physical and mental problems and results in one member leaving the family [39]. A study in Mexico reported that intimate partner violence has a significant impact on happiness and well-being [40]. The association between happiness and violence could be explained by common determinants of violence and life satisfaction [41].

People with higher income satisfaction were happier, although income had no direct correlation with happiness in the current study, which is in agreement with another investigation [42]. Happiness depends on the distribution of income in the community and satisfaction with it in comparison with that of others [43]. Income and happiness have a weak relationship, but the concept of income satisfaction could affect happiness, which is justifiable by the conceptual-referent theory of happiness [44].

A positive correlation between mental and physical health was seen in Sang Siah. Many studies have shown that physical and mental health are important factors which are associated with happiness [45]. Veenhoven says happiness could protect people against being ill, and mental and physical health affect each other [46]. In some studies of younger adults, however, no significant correlation between physical health and happiness was detected. Nevertheless, mental health had a strong association with happiness [47].

Religious people were happier than others not only in the current study but also in many others [45]. Religious beliefs give a sense of purpose to life [48] and provide the conditions for individuals to feel connected to an infinite source of power in times of unpleasant life experiences, fear, and sorrow [49].

The results of the current study show policy-makers that despite the economic problems caused by the sanctions against Iran, communities can be healthy and happy with the implementation of interventions to improve social support and strengthen familial foundations and safety in society, reduce violence, and increase job satisfaction and hope for the future (Additional file 2).

## Limitations of study

This study assessed happiness in the population under study through a cross-sectional study. It would be more conclusive if this study could be conducted prospectively to better clarify the causes and effects of happiness. Insufficient accuracy in answering the questions by some participants was another limitation of this study, but attempts were made to resolve this issue through the attention, patience, and training of the interviewers or by revisiting the participants at a better time.

The opportunity to survey the population of the entire city or nation and so gather data to study the variations did not present itself. Instead, WHO data on Iran from the previous year had to be used and compared.

## Recommendations

It is recommended that a larger longitudinal study be conducted to evaluate and compare happiness and its correlations in slums and other communities. Interventional studies in different communities could provide evidence in control setting by limiting bias. It would be necessary to design valid and reliable scales for evaluating happiness due to social, behavioral, economic, and cultural differences nationwide and to compare happiness levels in different clusters.

## Conclusions

Happiness in Sang Siah slum was correlated with social support and trust, safety in the neighborhood, hope for the future, job and income satisfaction, and facing violence. Marital status, smoking, and employment were other determinants of happiness in this population.

The present study provides policy makers with insight toward making a happy and healthy society through improving social support and trust between the people and the government, hope for the future, and preventing violence, crime, and insecurity in neighborhoods with intersectoral cooperation despite solving economic problems being the government’s primary goal.

## Supplementary Information

**Additional file 1.** Checklist of questions which were asked the participants to evaluate happiness and its correlates.

**Additional file 2.** General Health Questionnaire as a standard instrument for evaluating general health of participants.

## Data Availability

The datasets used and analyzed in the current study can be obtained from the corresponding author upon request. Data gathering: MRRH, MK, BH. Formal Analysis: STH, MRRH.  Technical advisors: BH, KBL, HJ. Technical management and supervision: KBL. Writing the original draft, manuscript, review and editing, final approval of the version to be published: all authors.

## Abbreviations

GHQ: General health questionnaire
OECD: Economic co-operation and development
GDP: Gross domestic product
SDG: Sustainable development goals
fSU: Former Soviet union
WHO: World health organization
