# Supplementary material for: Happiness in marginalized populations: a community–based study in South Central Iran
Source: BMC Psychol. 2021 Apr 23;9:58. doi: 10.1186/s40359-021-00545-2 (PMC8063348; doi:10.1186/s40359-021-00545-2)
Supplement: Supplementary file 1 — Additional file 1. Checklist of questions which were asked the participants to evaluate happiness and its correlates. [file 40359_2021_545_MOESM1_ESM.docx]

Appendix1:

Dear Mr. /Ms

In this way, you are invited to participate in “Happiness and its correlate in San-Siah “ research, which is carried out by the Health Policy Center of Shiraz University of Medical Sciences. The purpose of this research project is to investigate happiness and factors which are correlated with it to plan and do interventions in this community. You are free to participate or not in this research.
Before signing this consent form, make sure that you have noticed all the information in this form, also all your questions have been answered. If you have any other question you can contact with Principle investigator by contact number 00987132309615

***********************************************************************************

*Age:

*Gender: male / Female

*Education: Illiterate/ Primary school/ Diploma/ Associate degree And Bachelor/ Master and PHD

*Marital status: Single/ Married / Divorced/ Separated/ Widowed

*Number of children in family:

*Having job: Yes/No

*Job: unemployed/ Housewife/ student/ Daily worker/ Manual worker/ employee/ Manager/ Retired/ Disable

*Income/costs(income in compare with living costs):>1/<1/=1

*Migration from other country, other city or neighborhood duration of recent 5 years: Yes/ No

* Current Cigarette smoker (as smoking cigars on 1 or more of the 30 days before participation in a survey): Yes/ No

*Current Water Pipe smoker(as smoking cigars on 1 or more of the 30 days before participation in a survey) : Yes/ No

*Moderate Alcohol drinking(1 or more drinking in a day during last year) : Yes/ No

*Opium usage frequently 1 or more in a month in last year: Yes/ No

*Any kind of Insurance: Yes/ No

*Chronic diseases that you used medication for months or years or admitted in hospital because of that; Yes/ No

*Mental disease that you used medication or admitted in hospital because of that: Yes/ No

*Social support: do you have someone in family or friend that support you emotionally, physically or financially when you have problem?: Yes/No

*Bribe: Did you or your relatives and friends have noticed bribery by government officers in the past years? : Yes/No

*Violence: If you had been violent (physical, sexual and emotional) : Yes/No / place of violence(physical, sexual and emotional) home or outside

If you had been exposed to violence (physical, sexual and emotional) :Yes/ No / place of violence(physical, sexual and emotional) home or outside

*Happiness: “imagine a ladder, with steps numbered from 0 at the bottom to 10 at the top: The top of the ladder representing the best possible life and the bottom representing the worst possible life. which step of the ladder do you feel to stand at this moment?


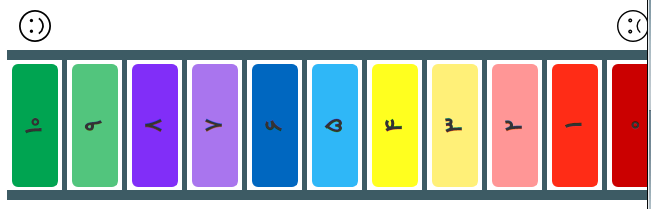


*Job satisfaction: “imagine a ladder, with steps numbered from 0 at the bottom to 10 at the top: The top of the ladder representing the best possible job and the bottom representing the worst possible job. Which step of the ladder do you feel to stand at this moment?


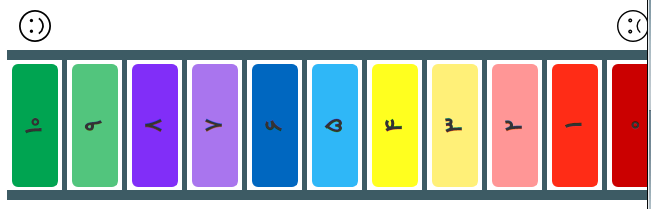


*Income satisfaction: “imagine a ladder, with steps numbered from 0 at the bottom to 10 at the top: The top of the ladder representing the best possible income and the bottom representing the worst. Which step of the ladder do you feel to stand at this moment?


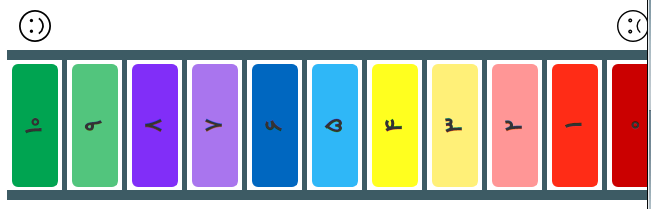


*Life satisfaction:

- In most ways your life is close to your ideal.
- The conditions of your life are excellent.
- You are satisfied with your life.
- So far you have gotten the important things you want in life.
- If you could live your life over, you would change almost nothing.

“imagine a ladder, with steps numbered from 0 at the bottom to 10 at the top: The top of the ladder representing the best possible life satisfaction and the bottom representing the worst. Which step of the ladder do you feel to stand at this moment?


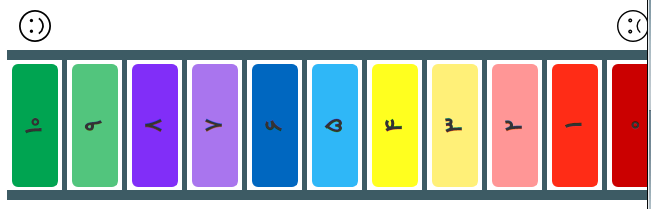


*Hope to the future: “imagine a ladder, with steps numbered from -5 to +5: that -5 representing the lowest hope to the future and +5 representing the highest.


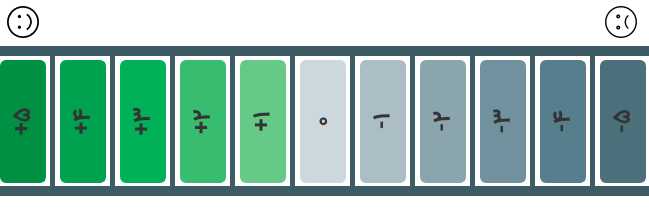


*Violence in neighborhood: according to crime in neighborhood that causes you feel unsecure, How much do you feel unsafe and insecure in neighborhood?


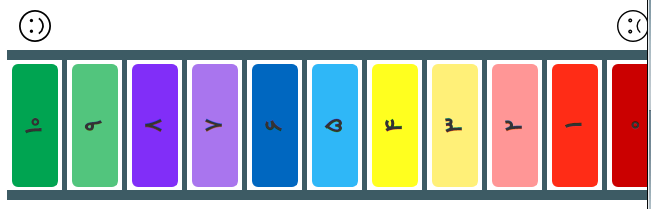


*Home satisfaction: How much do you feel satisfaction of your home?


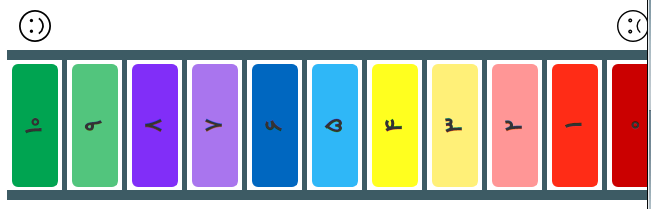


*Religious believes: do you belief that you are a religious people and do your religious tasks such as praying and fasting? : not at all/ sometimes/ Often/ Always/ completely

Refrence

1. Helliwell J, Layard R, Sachs J. World Happiness Report 2017. 2017. 2017.
