# Supplementary material for: Happiness in marginalized populations: a community–based study in South Central Iran
Source: BMC Psychol. 2021 Apr 23;9:58. doi: 10.1186/s40359-021-00545-2 (PMC8063348; doi:10.1186/s40359-021-00545-2)
Supplement: Supplementary file 2 — Additional file 2. General Health Questionnaire as a standard instrument for evaluating general health of participants. [file 40359_2021_545_MOESM2_ESM.docx]

**The 28-items of the scaled version of the GENERAL HEALTH QUESTIONNAIRE (Goldberg and Hillier 1979)**

HAVE YOU RECENTLY:

1. Been feeling perfectly well and in good health? not at all/ usually/ Always/ more than usual

2. Been feeling in need of a good tonic? not at all/ usually/ Always/ more than usual

3. Been feeling run down and out of sorts? not at all/ usually/ Always/ more than usual

4. Felt that you are ill? not at all/ usually/ Always/ more than usual

5. Been getting any pains in your head? not at all/ usually/ Always/ more than usual

6. Been getting a feeling of tightness or pressure in your head? not at all/ usually/ Always/ more than usual

7. Been having hot or cold spells? not at all/ usually/ Always/ more than usual

8. Lost much sleep over worry? not at all/ usually/ Always/ more than usual

9. Had difficulty in staying asleep once you are off? not at all/ usually/ Always/ more than usual

10. Felt constantly under strain? not at all/ usually/ Always/ more than usual

11. Been getting edgy and bad-tempered? not at all/ usually/ Always/ more than usual

12. Been getting scared or panicky for no good reason? not at all/ usually/ Always/ more than usual

13. Found everything getting on top of you? not at all/ usually/ Always/ more than usual

14. Been feeling nervous and strung-up all the time? not at all/ usually/ Always/ more than usual

15. Been managing to keep yourself busy and occupied? not at all/ usually/ Always/ more than usual

16. Been taking longer over the things you do? not at all/ usually/ Always/ more than usual

17. Felt on the whole you were doing things well? not at all/ usually/ Always/ more than usual

18. Been satisfied with the way you’ve carried out your task? not at all/ usually/ Always/ more than usual

19. Felt that you are playing a useful part in things? not at all/ usually/ Always/ more than usual

20. Felt capable of making decisions about things? not at all/ usually/ Always/ more than usual

21. Been able to enjoy your normal day-to-day activities? not at all/ usually/ Always/ more than usual

22. Been thinking of yourself as a worthless person? not at all/ usually/ Always/ more than usual

23. Felt that life is entirely hopeless? not at all/ usually/ Always/ more than usual

24. Felt that life isn’t worth living? not at all/ usually/ Always/ more than usual

25. Thought of the possibility that you might make away with yourself? not at all/ usually/ Always/ more than usual

26. Found at times you couldn’t do anything because your nerves were too bad? not at all/ usually/ Always/ more than usual

27. Found yourself wishing you were dead and away from it all? not at all/ usually/ Always/ more than usual

28. Found that the idea of taking your own life kept coming into your mind? not at all/ usually/ Always/ more than usual
